# Supplementary material for: Are carbon emissions trading and green financial instruments synergistic? -Comprehensive quantitative research based on content analysis
Source: PLoS One. 2024 Mar 7;19(3):e0298601. doi: 10.1371/journal.pone.0298601 (PMC10919601; doi:10.1371/journal.pone.0298601)
Supplement: S1 Data — (ZIP) [file pone.0298601.s001.zip › Data/地级市Z_F工作报告.zip!/║■▒▒/╛ú├┼╩╨/└┤╫╘╣1⁄2╓┌║┼í╛┬φ┐╦╩2╛▌═°í┐_2016.docx]

各位代表：

现在，我代表市人民政府向大会报告工作，请予审议，并请市政协各位委员和其他列席人员提出意见。

一、2015年和“十二五”时期工作回顾

刚刚过去的一年，是经济下行压力较大、困难较多的一年，也是荆门逆势而进、弯道超越的一年。我们在省委、省政府和市委的坚强领导下，在市人大、市政协的监督支持下，主动适应经济新常态，牢牢把握“竞进提质、升级增效”总要求，遵循“三维纲要”，坚持“投资第一、产业第一、工业第一”理念，深入实施中国农谷建设、柴湖振兴发展省级战略，经济社会发展态势良好，主要经济指标增幅位居全省前列。全年实现地区生产总值1388.46亿元，增长9.2%，增幅居全省第1位；规模以上工业增加值增长10.6%，居全省第1位；固定资产投资1404.58亿元，增长18.0%；社会消费品零售总额541.43亿元，增长13.3%，增幅居全省第3位；实际利用外资34000万美元，增长15%；出口总额88149万美元，增长14.8%；地方一般公共预算收入80.43亿元，增长19.3%，增幅居全省第3位；城镇常住居民人均可支配收入26731元，增长8.54%；农村常住居民人均可支配收入14716元，增长9.16%；居民消费价格涨幅1.6%；城镇登记失业率2.41%；人口自然增长率4.23‰；完成省定节能减排目标。我市成功创建国家森林城市，入选国家循环经济示范市，获批国家农业科技园区。

一年来，我们重点抓了以下工作：

（一）着力扩大有效投资。建立“中国农谷”、“柴湖振兴”、长江经济带等8大项目库，策划和储备“十三五”重点项目3619个，其中，424个项目纳入国家、省规划和项目库。强力推进“六个一”产业链招商，建立市、县（市、区）两级领导联系招商引资制度，分产业组建招商专班，成立7个驻点招商联络处，成功引进56个10亿元以上项目；全年利用市外境内资金1280亿元，增长14.3%。实行重大项目投资路线图管理，建立项目建设包联领导靠前指挥、项目秘书跟踪协调、驻点秘书脱产服务机制，全年实施亿元以上项目756个，其中324个项目竣工投产。强化政银企合作，加大项目融资力度，金融机构各项贷款余额794亿元，比年初增加141亿元，增长21.5%，增幅居全省第2位。

（二）着力推动产业转型。大力实施工业经济跨越式发展行动计划，编制并落实支柱产业发展规划，七大产业实现总产值3000亿元以上。在全省率先出台财政资金支持产业发展意见，组织开展“千名干部进千企、创优服务促发展”等活动，全市净增规模以上工业企业114家，规模以上工业增加值增幅连续10个月位居全省第一。制定加快传统产业结构调整促进转型升级的意见，实施技改项目628个，投资239.53亿元，荆门石化油品质量升级及适应性改造项目通过核准。战略性新兴产业加快发展，中航工业将重载飞艇、超轻型运动飞机研发制造等一批重大项目布局荆门。园区主导产业集群加快形成，重点工业园区实现工业总产值1851.59亿元，增长11.1%；荆门高新区总产值达到202.60亿元，增长13.7%，并获批国家体育产业基地。现代服务业发展水平提升，新增限上商贸单位102家、总部企业5家；成功举办2015荆门爱飞客飞行大会、秋游农谷紫薇花文化旅游节、第二届中国（京山）网球节和第八届油菜花旅游节，全年接待游客2500万人次，实现旅游综合收入130亿元，分别增长18%和21%。

（三）着力发展现代农业。启动中国农谷“三区三中心”建设，全面开展与中国农科院合作，推广“一高三新”面积200万亩，建成凯瑞百谷、联想佳沃等12个种业研发中心，一二三产业融合发展的“彭墩模式”在全省推广。强化柴湖振兴发展产业支撑，开工建设宏强百合、云天花海项目，建成农青园艺（一期）盆花基地，汽车锂电池等项目落户，吉象贝儿童装项目即将投产。推进农业产业化发展和标准化生产，粮食总产实现“十二连增”，新建畜禽标准化示范养殖场158个，水产品产量居全省第2位，省级以上农业产业化龙头企业达到71家，“三品一标”认证389个，“湖北神地”和“盛老汉”被认定为中国驰名商标，沙洋荣获“中国油菜籽之乡”称号。全市农机总动力达到456万千瓦，主要农作物耕种收综合机械化水平达到76%；实施土地整治41.3万亩，城乡建设用地增减挂钩项目6800亩；新改善灌溉面积30万亩；汉江以西水系连通及城市备用水源工程建设全面启动。

（四）着力统筹城乡发展。完成荆门市城市总体规划修编，大力开展“五城同创”，城市品质不断提升。加快漳河新区建设，漳河大道、爱飞客大道等建成通车。稳步推进旧城改造，拉通竹园路、果园一路等10条“断头路”，备受社会关注的海慧沟违法建筑全部拆除。深入开展经营秩序、运输抛撒、噪音油烟污染等9项专项治理，强力推进大气污染防治，中心城区淘汰黄标车2969辆，关闭燃煤锅炉49台，新增100辆清洁能源公交车，全市秸秆综合利用率达到89%。中心城区集中供热工程进展顺利，天茂集团入网试运行。完成基地造林16.3万亩，新建绿色通道1144公里，四旁植树827.5万株。汉江荆门港沙洋码头主体工程完工，漳河机场改扩建、蒙华铁路和枣潜高速荆门段、钟祥汉江二桥开工建设，汉江荆门港石牌码头、中心城区绕城公路建设加快推进，郑州—南宁高铁（襄阳—荆门—宜昌段）、荆门—荆州高铁正在开展前期工作。启动新型城镇化规划编制，推进新型城镇化示范带建设，后港被评为“全国文明镇”。

（五）着力抓好改革创新。深化行政审批制度改革，建立“三单一网”，公布涉企收费目录，实施第三批行政审批事项零收费。推进行政管理体制改革，精简87家事业机构，完成市直部门“大科室制”改革，单设市招商局，新设市创新创业服务局、市行政审批局；调整市区财政体制，推行财政专项资金项目库和清单管理制度。开展商事制度改革，实行企业登记“三证合一、一照一码”，企业登记提速86%，新增市场主体1.6万户。加快农村综合改革，完成1.04万平方公里农村集体土地所有权、3.5万宗宅基地使用权和集体建设用地使用权登记，按户连片耕种“沙洋模式”得到农业部充分肯定。创新投融资体制机制，设立产业发展基金、城市发展基金、创新创业引导基金和政银合作基金，与社会资本合作设立7支产业子基金；策划申报PPP项目110个，总投资1207亿元，竹皮河流域水环境综合治理（城区段）纳入财政部第二批示范项目；凯龙化工成为2015年全省首家在主板上市的企业，固润科技成功登陆“新三板”，四板挂牌企业新增35家。在全省率先出台支持大众创业、万众创新的“黄金三十条”，建成各类创新创业载体31个，入驻项目304个、创客1140人。

（六）着力增进民生福祉。民生投入占财政支出的比重达到79.8%，承诺的民生实事全部完成。开展“百个部门百家企业帮百村、万名干部扶万户”活动，组建210个扶贫工作队进驻所有贫困村，设立2亿元扶贫产业基金，实施精准扶贫项目124个，预计全年脱贫59907人。全市城镇新增就业52171人，组织城乡劳动者培训24194人次。建立“一门受理、协同办理”救助新机制，城乡低保标准提高10%，自付医疗费用救助比例提高到70%。对市特殊教育学校在校学生实行“四免”政策；资助家庭经济困难学生3.5万名。新建村级文化广场138个、农民体育健身工程350个，完成“户户通”安装6.5万户，“村村响”实现全覆盖。市青少年示范性综合实践基地投入使用。市一医南院、市中医医院住院部等一批重大公共卫生服务项目投入运行，市二医获批国家三甲综合医院。完成1%人口抽样调查。启动社区、乡村治理积分制试点工作。办理法律援助案件、事项1.36万件。完善社会治安动态防控“五为”机制，建立安全生产隐患排查治理标准化、数字化体系，人民群众的安全感和满意度持续提升。

作风建设事关政府形象。一年来，我们扎实开展政府系统“三严三实”专题教育，强化责任担当，争做“两为”干部，升腾发展气场，提升精神区位。加强法治政府建设，建立市政府常务会议学法制度，行政负责人出庭应诉率达到100%。主动接受市人大及其常委会的法律监督、工作监督，接受市政协的民主监督，办理人大代表建议140件、政协委员提案414件，满意率均达到98%以上。落实党风廉政建设主体责任，完善市级投资项目融资管理、政府投资工程监管、政府采购等制度，开展涉农资金管理使用、机关事业单位“吃空饷”、违规发放津补贴等专项整治。强化审计监督，深化政务公开，举办6期电视问政活动。坚持有腐必惩、有贪必肃，查处和曝光了一批突出问题。

各位代表，2015年各项任务的完成，为“十二五”时期的工作画上了圆满句号。过去的五年，全市人民团结拼搏、砥砺奋进，战胜前进道路上的各种困难，一步一个脚印，一年一个台阶，谱写了科学发展、跨越式发展的辉煌篇章！

这五年，是综合实力大幅提升的五年。主要经济指标保持中高速增长，增幅高于全省平均水平，走在同类城市前列。全市地区生产总值迈入“千亿俱乐部”行列，人均生产总值达到48042元，年均分别增长11.5%、11.3%。财政总收入突破“百亿大关”，地方一般公共预算收入突破80亿元，年均分别增长15.4%、28.2%。全社会固定资产投资累计完成额突破5000亿元，年均增长26.6%；社会消费品零售总额年均增长15.1%。发展地位不断提升，中国农谷建设、柴湖振兴发展上升为省级战略，我市被确定为全省唯一的国家节能减排财政政策综合示范市，荆门高新区跻身国家级高新区行列，东宝连续两年位居全省县域经济考核第一类第7名，京山、钟祥位居第二类前四强。

这五年，是产业结构不断优化的五年。全市三次产业比由19.9：48.4：31.7调整为14.5：52.5：33.0。工业总量紧随“一主两副”，居全省第4位，农产品加工成为“千亿产业”，化工产业总产值达到960亿元；规模以上工业增加值年均增长15.4%，规模以上工业企业由620家增加到1142家，其中，亿元以上企业由188家增加到528家。

农业现代化水平达到75%，农产品加工业总产值与农业总产值之比由1.52：1提高到3.41：1，农村居民人均可支配收入总量稳居全省第2位，京山、沙洋、钟祥连续多年被评为全省“三农”发展十强县（市），我市成为全省首个整市推进的国家现代农业示范区。第三产业增加值达到457.94亿元，是2010年的1.98倍。推动市校、校企合作，建成院士（专家）工作站33家，高新技术企业达到104家，高新技术产业增加值占地区生产总值比重达到12.7%，我市连续七届荣获国家科技进步先进城市称号。化工循环产业园是全省两个专业性化工园区之一，格林美电子废弃物循环利用工程技术研究中心成为行业内唯一的国家级中心，我市获批国家资源综合利用“双百工程”及“城市矿产”示范基地，预计单位生产总值能耗、二氧化碳排放量比2010年分别下降23%、23.8%，化学需氧量等主要污染物减排超额完成省定目标。

这五年，是城乡面貌深刻变化的五年。全市城镇化率从45.5%提高到53.8%。中心城区建成区面积从52.44平方公里扩展到65.73平方公里，连续两次夺得省城镇规划建设管理“楚天杯”。漳河新区“七纵九横”道路骨架全面拉开，被列为全省首批绿色生态示范城区。县（市）城、小城市、重点镇快速发展，京山通过国家生态县验收，钟祥获得“世界长寿之乡”称号；宋河等10个镇被评为全国重点镇，官垱被确定为全省“四化同步”示范试点镇；建设新型农村社区251个，江山被评为全国文明村，彭墩等48个村被命名为省宜居村庄，“幸福马岭”和“客店明灯”模式在全省推广。完成交通投资206亿元，是“十一五”时期的2.1倍；公路通车里程13914公里，等级公路比重达到96%，行政村通客车实现全覆盖；江汉运河建成通航，水运通航里程460公里。完成电网建设投资35亿元，是“十一五”时期的1.86倍。城市管理水平不断提升，全面推行数字化城管，建成“四管两中心”。关停葛洲坝城区生产线，城市生活垃圾无害化处理率达到100%，我市被评为全国节水型社会建设示范区、国家园林城市，加入国际低碳城市联盟。

这五年，是改革开放持续深化的五年。推进简政放权、放管结合，市级行政审批事项由365项减至148项，行政审批提速50%，行政审批零收费事项占比达到93%；公共资源交易规模比“十一五”时期增长2倍以上；京山建成全省首家县级公共检验检测平台；全市市场主体达到17.4万户，年均增长19.6%。流转农村土地230万亩，新型农业经营主体突破2万个，总数居全省首位，我市被确定为全省唯一的新型职业农民培育整市推进试点市。财政管理体制改革深入推进，金融改革取得新进展，新增银行类金融机构6家，达到18家，存款和贷款余额分别是2010年的2.1倍、2.56倍。国有企业改革步伐加快，国有资产运营效益进一步增强。对外开放程度不断扩大，累计实际利用外资13亿美元，完成出口31亿美元，年均分别增长18%、24%，中兴、联想控股、盈德、万达等大型企业落户我市，17家市内企业在境外投资办厂；海关、商检荆门办事处对外开办业务，全省第一家县级保税仓库——京山恒泰源保税仓库封关运营。

这五年，是群众生活切实改善的五年。城乡居民人均可支配收入增幅高于经济增速，年均分别增长11.8%、13.3%。全市累计新增就业23万人，比“十一五”时期增长64%。五大社会保险参保196万人次，我市被评为全国创业先进城市和技能振兴百佳城市。社会救助体系不断完善，累计救助城乡困难群众158万人次，发放救助资金14.23亿元。覆盖城乡的医疗卫生服务体系、公共卫生和医疗保障体系基本建立，在全省率先出台乡村医生生活补贴政策，公立医院综合改革实现县域全覆盖，“先看病、后付费”模式在全省推广。累计建成保障性住房2.4万套，改造棚户区（危旧房）5.5万户。解决61.64万农村人口饮水安全问题。投入11.8亿元，新建和改扩建学校、幼儿园419所，建设留守儿童服务站21个；义务教育均衡发展全域通过国家督导评估认定，普通高中教育质量居全省前列，职业教育服务地方发展能力不断提升，荆楚理工学院成为全省首批转型发展试点高校，我市荣获“中国教育创新奖”。文化资源信息共享工程基层服务点和农家书屋实现全覆盖，“农家乐杯”荣获“全国首届群众文化活动品牌”。基层社会管理和服务网络逐步健全，社区基础设施达标率90%，村级综合服务社总数达到1206家。人口计生工作连续两年获得全省党政领导线考核一等奖。安全生产形势持续平稳，食品药品安全监管不断加强，应急管理体系更加健全。完成“六五普法”任务，法治荆门和平安荆门建设取得明显成效。国防动员和国防后备力量建设稳步推进，优抚安置、双拥工作成效显著。民族宗教、外事侨务、对台、民防、防震减灾、援疆援恩等工作取得新成绩，机关事务、住房公积金、物价、移民、气象、档案、地方志、保密、新闻出版等工作迈上新台阶，妇女儿童、青少年、老龄、残疾人保障、红十字、慈善等事业实现新发展，多个单位、部门荣获全国、全省荣誉和表彰。

各位代表！回首过去的五年，我们深切地感到，荆门取得的巨大成就，是省委、省政府和市委正确领导的结果，是全市人民团结奋斗的结果，是社会各界大力支持的结果。在此，我代表市人民政府，向全市人民，向人大代表、政协委员，向各民主党派、工商联、人民团体、无党派人士、离退休老同志，向驻荆部队、武警官兵，向“十二五”时期与我们并肩战斗的老领导、老同事，向所有关心支持荆门发展的同志们、朋友们，表示衷心的感谢并致以崇高的敬意！

成绩来之不易，经验弥足珍贵。五年的发展启示我们，必须始终坚持把加快发展作为第一要务，以等不起的紧迫感“缩差领跑”，以慢不得的危机感“弯道超越”，以坐不住的责任感适应新常态。必须始终坚持把转型升级作为经济发展的主攻方向，调整存量、做优增量，努力实现更高质量、更有效益、更加公平、更可持续的发展。必须始终坚持把改革创新作为加快发展的根本动力，在深化改革、扩大开放、加快创新中按下“快进键”，展现新作为。必须始终坚持把以人为本作为各项工作的出发点和落脚点，努力让人民群众生活得更加富裕、更加安全、更有保障、更有尊严。

在总结成绩的同时，我们也要清醒地看到，全市经济社会发展中还存在着不少困难和问题，主要表现为：经济总量不大，产业结构不优，项目支撑不够，扩大经济规模、调整经济结构任务繁重；创新驱动能力不足，民营经济发展不够，企业核心竞争力、抗风险能力不强，培育壮大龙头企业、提升经济活跃度任务繁重；城市能级不高，交通基础设施不完善，提升城市承载能力任务繁重；关系群众切身利益的环境保护、扶贫开发、公共服务等方面还存在不少薄弱环节，保障和改善民生任务繁重；政府系统干部的思想作风、能力水平还存在一些不足，加快建设法治政府、服务型政府任务繁重。对此，我们一定高度重视不回避，勇于担当不退缩，采取有效措施加以解决，决不辜负全市300万人民的期望！

二、“十三五”时期经济社会发展的总体要求

各位代表，“十三五”时期，是荆门提前全面建成小康社会的决胜期，是全面深化改革的攻坚期，是爬坡过坎、跨越赶超的关键期。根据市委《关于制定荆门市国民经济和社会发展第十三个五年规划的建议》，市政府制定了《荆门市国民经济和社会发展第十三个五年规划纲要（草案）》（以下简称《纲要草案》），提请大会审议。

“十三五”时期全市经济社会发展的指导思想是：高举中国特色社会主义伟大旗帜，全面贯彻党的十八大和十八届三中、四中、五中全会精神，以马克思列宁主义、毛泽东思想、邓小平理论、“三个代表”重要思想、科学发展观为指导，深入贯彻习近平总书记系列重要讲话精神，紧紧围绕“四个全面”战略布局，牢固树立创新、协调、绿色、开放、共享的发展理念，切实把握“三维纲要”，深入实施“两大战略”，坚持生态立市、产业强市、资本兴市、创新活市，统筹推进经济建设、政治建设、文化建设、社会建设、生态文明建设，确保提前全面建成小康社会，打造江汉平原中心城市、湖北新兴增长极、全国新型城镇化和城乡一体化示范区。

今后五年全市经济社会发展的总体目标是：综合实力迈上新台阶，主要经济指标增幅高于全省平均水平、高于同类城市，力争2020年全市地区生产总值突破2200亿元，地方一般公共预算收入超过160亿元，户籍人口城镇化率达到40%以上。人民生活水平和质量显著提高，贫困人口2018年全部实现脱贫，城镇居民人均可支配收入达到全国平均水平，农村常住居民人均可支配收入保持全省领先，覆盖城乡的基本公共服务和社会保障体系更加完善。基础设施明显改善，交通、水利、能源、信息网络等建设取得较大进展。生态文明建设成效明显，主体功能区布局和生态安全屏障基本形成，绿色低碳的生产方式和生活方式基本普及，资源节约、集约和循环利用水平不断提高。社会文明程度大幅提升，中国梦和社会主义核心价值观更加深入人心，文化软实力明显增强，民主法治更加健全。改革开放取得重大进展，重点领域和关键环节改革取得实质性突破，开放型经济体系基本形成。《纲要草案》从经济发展、创新驱动、生态环境、公共服务、人民生活五个方面，提出了“十三五”时期经济社会发展的41个主要目标，形成了较为完整的目标体系，力求体现科学发展、转型发展的新要求。

“十三五”时期，我们要以五大发展理念为引领，抢抓机遇、克难奋进，努力将“提前全面小康”变成惠及全市人民的美好现实，奋力实现荆门经济社会发展新跨越。

（一）推进创新发展，加快经济发展动力转换。坚持把发展基点放在创新上，加快创建国家创新型试点城市，建成江汉平原区域科技创新中心、国家现代农业科技创新中心。围绕释放新需求、创造新供给，推动投资、消费、出口协同发力。加强供给侧结构性改革，提高供给体系质量和效益。深入实施创新驱动发展战略，培育一批有竞争力的创新型领军企业，建设一批产业技术创新联盟和院士工作站，推动荆门高新区创建国家创新型特色园区；深入推进大众创业万众创新，构建“种子—苗圃—孵化器—加速器—产业园”的梯级孵化体系。高举中国农谷旗帜，按照“一谷双核、多点支撑”布局，加快建设“三区三中心”，在全省率先转变农业发展方式、率先基本实现农业现代化，成为国家现代农业示范区的标杆、国家农业科技园区的标杆。深入落实《中国制造2025》，强力实施工业经济跨越式发展行动计划，培育壮大七大支柱产业，建成全国新型能源化工基地、全国生态农产品加工基地和国家循环经济示范城、国家健康产业城、国家通用航空产业城。突破性发展现代服务业，打造区域性金融保险服务中心、江汉平原生产性物流基地、国内知名的运动休闲旅游目的地。统筹全市产业布局，建设20个左右特色产业园区，支持各县（市、区）培育2—3个百亿特色产业集群。实施“互联网+”行动计划，建成10个“互联网+”产业园。构建充满活力的体制机制，确保行政管理体制和行政审批制度改革、投融资体制改革、农村综合改革等走在全省前列。

（二）推进协调发展，增强发展整体性。推动中心城区与各县（市、区）协调发展，加快中心城区经济业态和城市形态转型升级；支持钟祥争创全国百强县市，京山争当全省县域经济发展“排头兵”，沙洋进入全省县域经济“第一方阵”，屈家岭管理区成为全国转变农业发展方式示范区。提高新型城镇化发展质量，构建以中心城区为核心、三个县域城市为支撑、五条城镇带为纽带的“一主三副五带”全域城镇化格局；加快漳河新区建设，建成城市新中心和绿色生态新城；推动中心城区与钟祥相向发展，打造荆钟组合都市区，形成100万人口的大城市；规划建设一批特色城镇、新型农村社区、新农村示范村和美丽乡村。加快推进“新五化”，促进城乡一体化发展。构建现代化基础设施体系，初步建成江汉平原城市群区域性综合交通枢纽，构建现代水利设施网络，完善信息基础设施，提升能源保障能力。提升文化软实力，建设全省文化强市。推动军民融合深度发展，实现生产力发展与战斗力提高相互促进。

（三）推进绿色发展，建设生态文明家园。树立全域生态理念，编制生态立市总体规划和主体功能区规划。建设汉江生态保护带，发挥荆山、大洪山的屏障作用，保护漳河、惠亭、温峡、长湖等湿地资源，打造山水林田湖生命共同体。大力发展绿色经济，强力推进节能减排，促进资源节约高效利用，深化国家循环经济示范市建设。实行最严格的环境保护制度，加强水生态保护，加大大气污染治理力度，提升土壤环境质量，巩固和深化国家森林城市建设。推动生产方式、生活方式和消费模式绿色转型，完善绿色发展体制机制，在全省率先建成国家生态文明试验区。

（四）推进开放发展，构建开放型经济格局。主动对接国家“一带一路”战略，推进铁路网与渝新欧、汉新欧、郑新欧等国际物流线路的对接与互通；建设汉江荆门港，与武汉新港、宁波港等合作，融入海上丝绸之路；积极争取建设综合保税物流中心，加快打造荆门陆地港、沙洋监管码头、国际产业园区。创新招商方式，提升“引进来”的质量和水平。加快“走出去”步伐，形成参与国际分工的荆门方阵。深化区域战略合作，促进城市发展互惠互利。营造与国际接轨的营商环境，不断提升参与国际国内经济合作竞争的能力和水平。

（五）推进共享发展，不断增进人民福祉。按照“精准扶贫，不落一人”的要求，大力实施脱贫攻坚工程，确保2018年贫困人口全部脱贫、贫困村脱贫全部“销号”、贫困乡镇脱贫全部“摘帽”。整体推进柴湖精准扶贫、精准脱贫，努力将柴湖打造成为城乡统筹示范区、移民发展样板区、民生改善先行区。大力促进教育公平，推动义务教育均衡发展，全面普及学前教育和高中教育，打造全国职业农民培训基地，建设江汉平原职业教育中心，支持荆楚理工学院打造全国知名的应用型大学。实施更加积极的就业政策，努力实现更加充分、更高质量的就业。完善社会保险体系，落实全民参保计划，基本实现法定人员全覆盖。开展健康荆门建设，打造江汉平原健康卫生服务中心。坚持计划生育基本国策，积极开展应对人口老龄化行动，保障妇女和未成年人权益，支持残疾人事业发展。围绕建设平安荆门，构建共建共享的社会治理格局。

各位代表，“十三五”蓝图已经绘就，跨越式发展的号角已经吹响，让我们与全市人民一道，团结拼搏，开拓创新，闯出改革发展的新天地。我们坚信，“十三五”期末，一座实力强、百姓富、生态美的新荆门必将崛起在荆楚大地上！

三、2016年主要工作

2016年是实施“十三五”规划的开局之年，是推进结构性改革的攻坚之年，是提前全面建成小康社会的关键之年。做好今年的工作，意义深远，责任重大。

今年全市经济社会发展的主要预期目标是：地区生产总值增长10%左右；规模以上工业增加值增长11%；固定资产投资增长20%；社会消费品零售总额增长13%；地方一般公共预算收入增长12%；实际利用外资增长8%；出口总额增长11%；城镇常住居民人均可支配收入增长10%，农村常住居民人均可支配收入增长10.5%；居民消费价格涨幅控制在3.5%以内；人口自然增长率7‰以内；城镇登记失业率控制在4.5%以内；节能减排完成省定目标。

做好2016年工作，必须全面贯彻落实中央和全省经济工作、城市工作会议以及市委七届八次全会精神，坚持“稳中求进”总基调，坚持“竞进提质、升级增效、以质为帅、量质兼取”总要求，坚持“投资第一、产业第一、工业第一”理念，深入实施“两大战略”，着力加强结构性改革，推动供需两侧共同发力，更加注重质量效益，更加注重创新驱动，更加注重绿色发展，更加注重对外开放，更加注重公平公正，奋力实现“十三五”良好开局。重点抓好以下工作：

（一）夯实工业主导地位

积极应对经济下行压力，推动实体经济健康平稳发展，努力实现投资有回报、产品有市场、企业有利润、员工有收入、政府有税收、环境有改善。

提升产业竞争力。加快化工、机械、建材、农产品加工等传统产业转型升级，实施“百企技改”行动，支持企业“二次创业”。将技改项目视同招商项目，采取财政贴息、政府购买中介组织服务等方式，加大政策支持力度，打造技改示范企业20家，力争技改投资增长23%。培育壮大战略性新兴产业，深化与中航工业合作，突破性发展通用航空产业；发挥京山轻机、中集宏图等企业示范作用，加快发展智能装备制造产业；用好荆门高新区国家体育产业基地、武汉国家生物产业基地荆门产业园“金字招牌”，大力发展大健康产业；支持格林美等重点企业发展，扩大再生资源利用与环保产业规模；推动金泉新材料等企业做大做强，构建新能源新材料产业体系；依托华工科技荆门光电子信息产业园、东宝电子信息产业园、大柴湖德同科技产业园，积极发展电子信息产业，力争战略性新兴产业增加值增长20%。大力发展高新技术产业，全市新增高新技术企业25家，高新技术产业增加值占地区生产总值比重达到13.2%，研究与试验发展经费占比达到1.5%。

增强企业发展实力。按照“优化存量、引导增量、主动减量”要求，对企业分类建档立卡，实行一企一策。继续推行市、县（市、区）领导包联龙头企业和部门联系骨干企业制度，建立重点税源企业清单，实行财政调度资金、财政贴息资金、政银合作基金向优质企业集中，力争新增规模以上工业企业100家以上，新增百亿企业1家、50—100亿元企业3家、10—50亿元企业10家。坚持“企业主体、政府推动、市场引导”原则，鼓励产能过剩企业和困难企业兼并重组；支持京山轻机、天茂、新洋丰、凯龙等企业“走出去”发展，形成具有较强竞争力的大型企业集团。对于连续多年亏损，产品确实没有市场，没有企业看中又严重资不抵债的企业，依法破产清算。支持企业开拓国内外市场，推动产业链上下游市内企业供需对接，加大政府对本地产品的采购力度；新增出口企业10家，培育出口额过千万美元的企业3家。引导企业加强管理创新、制度创新和科技创新，力争商标注册量达到7000件，实现入围中国名牌价值500强零的突破。

建设特色产业园区。加强基础设施建设，配套学校、医院、商业等公共设施，构建研发设计、综合物流、公共检验检测和金融服务平台，完善园区功能，增强园区承载能力。围绕培育园区主导产业，优选龙头企业，以政府投融资平台为主体，向银行申请中长期贷款，用于购置重资产，企业通过租赁或回购方式取得使用权或产权。强力推进“园办一体化”改革，推动行政审批和中介服务机构到园区集中办公，实行一站式服务。完善园区主导产业发展规划，对新入驻企业、新上项目，严格按照功能分区入园，引导产业集聚集群发展，力争重点工业园区工业总产值占全市工业总产值的比重达到60%，园区主导产业产值占园区工业总产值的比重达到45%。

全力服务实体经济发展。加强煤电油运、资金、用工、用地等要素保障，着力为企业排忧解难。扎实开展降低实体经济企业成本行动，严格落实国家降低制造业增值税税率、企业社保缴费率、大工业电价、物流费用等一系列政策；全面清理行政审批中介服务项目，放开市场准入，加快推进中介机构脱钩改制工作，坚决整治“红顶中介”；完善涉企收费清单，实行明码标价，清单外的一律不得收费；开展金融机构不规范收费专项治理活动，严禁违规收费或搭车收费，政府独资或控股的融资性担保公司，收取工业企业担保费率一律降到1%；对企业新录用人员，由政府认定的就业培训机构开展岗前培训，给予财政补贴。确定100家重点企业作为负担监测点，建立企业负担督办检查、定期通报和责任追究机制。

（二）持续推进农业增效、农民增收

以转变农业发展方式为主线，加快构建现代农业产业体系、生产体系、经营体系。

提高农业综合生产能力。坚持最严格的耕地保护制度，落实藏粮于地、藏粮于技战略，全面实行永久性基本农田划定，创建耕地保护机制创新先行区；深入开展粮油高产示范创建、中低产田改造和高标准农田建设，实施土地整治20万亩，完成5.2万亩土壤修复任务。加强农田水利建设，继续抓好汉江堤防整治和水库除险保安，清淤、衬砌渠道2800公里，新建、改造泵站300座，整治塘堰3500口，改善灌溉面积30万亩。确保院士村项目建成投入使用，深化与中国农科院等科研院所合作，推动科技成果应用和转化。加快荆门农用航空服务站建设，力争全市农作物耕种收综合机械化水平达到77%。加强农村基层科技服务体系建设，推广一站式服务，办好“12316三农”综合信息服务平台。

发展生态高效农业。推广应用“香稻嘉鱼”等10种生态种养模式，新建标准化畜禽养殖示范场80个、现代渔业示范园区5个、林业特色产业基地6个，全市“一高三新”面积突破220万亩。突出生态、富硒、长寿等元素，加强省级富硒示范基地建设，在每个县（市、区）集中建设5万亩生态农业示范区，新认证“三品一标”30个。大力发展绿色、循环农业，打造粮油转化、秸秆综合利用、农业废弃物循环利用产业链，力争秸秆综合利用率达到90%。实施“互联网+智慧农谷”行动计划，建成中国农谷大数据中心。加强市、县农产品质量安全追溯监管平台建设，推进农产品质量安全县（市）创建，确保群众“舌尖”上的安全。

推动一二三产业融合发展。推进中国农谷现代种业科技园建设，建成杂交油菜、马铃薯、甘薯良种研发和繁育基地。依托中国农科院（华中）农产品加工成果转化示范基地、各地农产品加工园区，推动农产品加工向精深化、功能化、品牌化方向发展，力争农产品加工业产值增长14%，与农业总产值之比达到3.6：1。鼓励发展订单生产、连锁经营、直供配送，加强冷链物流体系和农业电子商务建设。积极发展休闲农业、观光农业，启动建设屈家岭遗址公园；做大做强柴湖盆花基地，打造“中国花城”。抓好彭墩“1+9”示范区建设，推广复制“彭墩模式”；支持沙洋与神州数码合作，打造全国三产融合发展示范县。

创新农村经营机制。发展壮大新型农业经营主体，新增省级以上农业产业化龙头企业5家，创建省级以上农民专业合作社40家。探索实行“合作社+龙头企业+农户”的土地规模经营模式，引导农民入社、土地入股，新建土地股份合作社50家。完善农村公共服务“以钱养事”机制，推行政府购买农业公益性服务；依托新型农业经营主体，建立“三农”服务中心；加快发展航空植保、气象预警防灾、农业保险、田间管理等经营性、公益性服务。推广“粮食银行”模式，开展粮食流通运作“四代一换”试点。

（三）开展加快发展现代服务业行动

加快建设省级现代服务业示范区和服务业综合改革试点区，力争服务业增加值占地区生产总值比重达到33%。

大力发展现代物流业和商贸服务业。编制中心城区现代物流业发展规划，加快荆门综合物流园和各县（市、区）物流园建设，积极发展第四方物流。着力培育壮大物流企业，新增4A级以上物流企业1家、3A级物流企业4家。发挥大型商贸流通企业、城市综合体、城市商圈的带动作用，推进特色商业街区建设。建立健全农村商贸网点和配送体系，加大农产品大市场等专业市场建设力度。推动电子商务进企业、进社区、进农村，建成人民万福、中兴农谷等电商产业集聚区，实现电商服务站点行政村全覆盖，孵化培育电商企业和网店1000家，力争电商交易额增长40%。

大力发展文化体育旅游业。规划建设积分制管理文化创意产业园、通用航空文化产业园。推动旅游景区提挡升级，支持明显陵、彭墩、绿林山创建国家5A级景区。探索建立旅游年卡。加强与周边城市合作，合力开拓大洪山生态旅游市场，高标准打造一批精品旅游线路。高质量举办第十二届中国（荆门）菊花展览会、第二届荆门爱飞客飞行大会暨航空旅游节、第七届亚洲赏鸟博览会、第九届中国农谷荆门（沙洋）油菜花旅游节等节会活动，力争全年接待游客2850万人次，实现旅游综合收入150亿元以上。

大力培育服务业新业态。支持中航工业医养结合基地、漳河新区高端养老社区、市中医医院和康复医院康复养生护理中心建设，提供多样化健康服务。围绕爱飞客镇规划布局，积极发展航空运营与服务、航空培训、航空体验、航拍航摄、测绘勘探等服务业，启动航空大世界、飞行体验中心等项目建设。深化与神州数码、腾讯、软通动力等企业合作，推进市民卡建设，构建新型市民融合服务体系。鼓励发展会展经济，加快建设中国农谷农产品会展中心、爱飞客综合体会展中心。积极发展社区服务、工业设计、科技服务、商务咨询、检验检测等服务业态。

（四）做大做实投资底盘

把招商引资和项目建设作为经济工作的主抓手，以增量优化带动存量调整，增强经济发展的支撑和后劲。

强力推进招商引资。坚持引龙头、强链条、补短板，强化产业链招商。创新招商引资方式，推行股权招商、委托招商、以商招商，引进一批产业层级高、投资规模大和带动能力强的项目。巩固“珠三角”招商引资阵地，积极承接“长三角”地区产业转移，加快与上海共建产业园区。加强与央企合作，力促与中航工业合作的重点项目落地。大力引进外资，寻求国际战略投资者来荆投资。筹建各省（市）荆门商会，召开首届荆门楚商大会。完善招商引资体制机制，强化目标责任考核，力争引进世界或国内500强企业4家、企业区域性总部3家；引进项目400个，其中，5亿元以上项目60个；招商引资到位资金增长25%以上。

狠抓重大投资增长点。深化项目建设大竞赛活动，实施“千亿项目投资工程”，统筹推进650个亿元以上项目建设，总投资4455亿元，年度投资1000亿元以上。在重大产业项目方面，开工建设荆门石化280万吨重油催化裂化装置、博世电梯、新冶集团人造蓝宝石长晶、粤创集团6代半液晶显示模组等项目，加快金泉新材料动力与储能电池、格林美动力电池材料前驱体扩建、君尚科技医药中间体等项目建设，确保荆门石化200万吨渣油加氢装置、洋丰中磷60万吨硝基复合肥、葛洲坝异地技改、福耀玻璃三期扩建、台顺集团工业机器人等项目建成投产，力争今年预增亿元产值的工业增长点达到150个以上。在重大交通项目方面，加快蒙华铁路荆门段建设，推动荆门—荆州高铁前期工作，力促郑州—南宁高铁（襄阳—荆门—宜昌段）开工；抓好枣潜高速荆门段和钟祥汉江二桥、沙洋汉江二桥、中心城区绕城公路、中心城区—石牌及东桥—子陵等一级公路建设；加快汉江荆门港、漳河机场建设，做好冷水机场改扩建前期工作。在重大水利项目方面，抓好汉江以西水系连通及城市备用水源工程建设，做好鄂北水资源配置二期（荆门部分）、荆门汉东引水工程前期工作，完成三干渠灌区配套与节水改造年度建设任务。在重要民生项目方面，推进农村和城市电网改造，提升用电保障能力；推动“光纤入户”“宽带下乡”工程，基本实现城镇家庭宽带接入能力达到20兆/秒以上，行政村及以上4G无线网全覆盖、宽带网络全部通达；开工建设市民之家和“四馆四中心”。

加强项目建设全过程管理。按照有责任主体、投资主体、时间节点、进度安排、路线图计划的“五有”要求，做好项目前期工作。抓住项目规划、安评环评和招投标等关键环节，加快项目推进进程。坚持“以人为本、依法补偿、贴心安置、长效帮扶”原则，突破征地拆迁难点，为项目落地创造条件。落实项目建设领导包联制、秘书驻点制、季度拉练制、全程督查制，实行集中开工、集中竣工投产制，全力推动项目快开工、早见效。

（五）大力推进新型城镇化和城乡一体化

坚持区域协同、城乡一体、产城融合，着力构建城乡统筹发展新格局，力争常住人口城镇化率达到55.3%，户籍人口城镇化率达到33.5%。

强化规划引领作用。启动编制荆门2049城市远景发展战略规划，为建设江汉平原中心城市提供规划支撑。加快中心城区控制性详细规划修编，强化重点区域控规编制和城市设计、城市修补，完善各项专项规划。推进镇区规划编制，加快牌楼—石牌、掇刀—沙洋、京山—屈家岭3条新型城镇化示范带建设，提升宋河、钱场、后港、胡集、柴湖、易家岭6个小城市建设水平，整镇推进孙桥、石牌、官垱、牌楼、团林、漳河、长滩7个新型城镇化试点。以新型农村社区和美丽乡村规划为引领，创建10个省级宜居村庄、70个省级绿色示范村。

统筹推进新区建设和老城区改造。着力完善漳河新区基础设施，开工建设荆楚大道、荆山大道等31条主次干道，确保天鹅路、天山路等7条道路建成通车；加快建设北片区地下综合管廊，完成泉口换热站至双喜片区供热管网铺设；实施“五湖连通”工程，推进凤凰湿地公园、蒙泉生态园、杨柳公园、荆西公园建设。着力强化漳河新区产业支撑，启动绿色生态科技产业城项目，开工建设双仙总部经济园、华中汽车金融博览中心，加快中央商务区建设，确保国华人寿、万达广场、红星国际广场建成投入使用。提升老城区宜居水平，实施象山大道和中心城区三条高速公路出入口综合整治；按照“两减两增”原则，加快胡祠堂、惠民市场、汉正街片区等15个棚户区和老旧住宅小区改造。支持各县（市）建设新区，推动京山温泉新区与京山经济开发区、沙洋滨江新区与沙洋经济开发区、钟祥南湖新区与大柴湖经济开发区融合发展。

进一步提升城市管理水平。深入开展“五城同创”，成功创建省级环保模范城市，获得全国文明城市提名资格。完善市、区城市管理体制，构建城管综合执法联动机制，推行第三方考评。选定违建存量较大的片区，实行集中整片拆除。加强出店经营、散装货场、运输抛撒、马路市场等专项整治，力争再成功创建1—2条省级市容环境美好示范路。进一步提高城市道路机械化作业覆盖率和人工保洁质效，强化“门前三包”管理。大力实施“互联网+城市管理”行动，依托电子政务云平台，统筹推进智慧城管、智能交通等服务。

（六）加快生态荆门建设

牢固树立“绿色决定生死”理念，认真落实《关于坚持生态立市建设生态荆门的决定》，既要金山银山，也要绿水青山，努力把荆门建成绿色低碳、和谐宜居的生态城市。

加大环境治理和生态保护力度。加强大气污染综合治理，加快推进中心城区集中供热，彻底关停燃煤锅炉；关闭城市规划区范围内所有的煤场、沙场、石膏矿；实施国电长源、华能热电联产超低排放改造；加快葛洲坝老厂区矿山植被恢复。以最严格的措施保护漳河、温峡、惠亭等饮用水源，深入推进竹皮河、浰河、西荆河等流域污染治理，加强长湖流域面源污染防治和生态修复。实施农村生态环境综合治理“八大行动计划”，推广“客店明灯”模式，创建省级以上生态乡镇6个。实施城市垃圾分类回收，推进生活垃圾无害化处置和建筑垃圾资源化利用。深入开展“绿满荆门”行动，完成植树造林12万亩。

推进资源能源节约循环高效利用。实行能源和水资源消耗、建设用地等总量和强度双控行动，推进企业节能、节水改造，加大工矿废弃地复垦利用力度，积极创建国土资源节约集约模范县（市），力争亿元生产总值地耗下降4%。严格项目评审，严禁新上高耗能、高污染、高排放项目。围绕石化、磷化、建材、“城市矿产”等重点行业和重点领域，加快荆门化工、荆襄磷化、格林美等特色循环产业园建设，推进企业循环式生产、产业循环式组合、园区循环式改造。

倡导绿色生活方式。构建城市慢行系统，新增新能源公交车100辆、城市绿道16公里。推广节能和绿色建筑，新增节能建筑面积140万平方米、绿色建筑20万平方米。统筹推进绿色机关、绿色企业、绿色家庭等创建活动。

（七）扎实推动改革措施落地

充分释放改革红利，力争在重点领域和关键环节取得新突破。

深化行政管理和行政审批制度改革。健全政府权力清单、责任清单和企业投资负面清单、财政专项资金项目库和清单管理制度。完善行政审批事项目录清单管理办法，确保目录之外无审批；全面推行“一口受理、一站办结”，落实“三集中、三到位”，推动市级行政审批向市行政审批局集中；进一步优化审批流程，实现行政审批服务再提速10%以上。整合市级公共资源交易平台，促进公共资源交易规范化和电子化。加快市检验检测产业园和市县公共检验检测机构建设。探索实行市、县综合执法，扩大乡镇综合执法试点范围。

深化投融资体制机制改革。做大产业发展母基金规模，拓展子基金种类，组建通用航空等子基金，围绕支柱产业链条，构建政府引导、基金参股、特色鲜明的基金投资体系。推进政府投融资平台转型发展，将融资平台存量项目转型为PPP项目，组建市投资控股集团。实施市域、县域金融工程，建立市、县金融服务中心，开展金融服务网格化工作。推动政银企深度对接，做大做强政府出资或控股的中小企业担保公司，扩大贷款风险补偿基金规模，引导金融机构创新金融产品、加大信贷投放力度，力争新增贷款180亿元以上。构建上市后备企业服务平台，推动更多优质后备企业在主板、中小板、创业板、“新三板”、四板直接融资，力争新增“新三板”挂牌企业15家、四板挂牌企业30家。

深化农村综合改革。推广按户连片耕种“沙洋模式”，解决农村承包土地分散化问题，促进适度规模经营。落实土地所有权、承包权、经营权分置政策，完成农村宅基地和集体建设用地使用权确权登记。全面启动不动产统一登记。深化赋予农民对集体资产股份权能改革试点工作，争取全国农村承包土地的经营权和农民住房财产权抵押贷款试点申报成功。

深化其它领域改革。以加强国有资产监管、完善法人治理结构、优化产权结构为重点，深化国有企业改革。深入推进事业单位分类改革、养老保险制度改革、市直行政事业单位国有资产管理运营体制改革，完成公务用车改革。创新财政性资金使用方式，完善政府举债融资机制，提高科学理财水平。积极探索推进农业转移人口市民化新机制，推动户籍制度改革和居住证双落地；开展宅基地改革和土地承包经营权有偿退出试点。创新保障性住房供应方式，探索建立“房票”制度；把政府收购商品房作为保障性住房主要房源，确保货币化安置比例超过50%；推行“市场租金、租补分离、分类补贴”，将符合条件的新市民纳入住房保障范畴。

深入推进大众创业万众创新。按照“一空间一产业”原则，推动众创空间市场化、专业化、集成化、网络化发展，开展“大学生走进众创空间行动”，打造“荆门龙泉汇”路演品牌，创建国家级众创平台1家、省级3家，新增创客1500人。探索建立柔性引才机制，力争全年引进200名高层次人才。创新市校、校企合作方式，新建省级以上工程技术研究中心、企业技术中心10家以上，新增发明专利授权量80件。完善“荆创贷”等金融产品，开展科技创新券试点，加快组建市级创业资本园。进一步降低创新创业政策门槛，扩大受益面，强化对政策执行情况的督查和评估。

（八）切实保障和改善民生

制定并实施改善民生“三年行动计划”，着力保障基本、创新机制、强化治理，以改善民生的实际成效取信于民。

扎实打好脱贫攻坚战。完善市、县（市、区）、乡（镇）、村总体扶贫规划、专项扶贫规划和行业扶贫规划。按照“六个精准”要求，加快“五个一批”工程建设，确保易地搬迁扶贫1793户、低保和“五保”人均补贴提高1000元、每个县（市）投放扶贫小额贷款5000万元以上，建设5个脱贫示范乡镇、10个脱贫示范村、300个脱贫示范户。

做好就业和社会保障工作。全面落实就业创业扶持政策，确保城镇失业人员再就业8000人，困难人员再就业3500人。新增五大社会保险参保1万人次。完善临时救助制度，提高临时救助封顶线。建成城市社区居家养老服务中心24个、农村老年人互助照料中心64个、留守儿童服务站12个。加大对0—14岁残疾儿童康复救助力度。

提升公共服务水平。完成中心城区义务教育学校管理体制调整。加快建设荆楚职业学院，推动建设荆门足球学校，支持荆楚理工学院筹办通用航空学院、积分制管理商学院等。改善基层卫生服务条件，加快推进分级诊疗。加强健康管理，建立覆盖生命周期的健康服务新模式。落实“全面二孩”政策，综合治理出生人口性别比。开展文化惠民及送戏下乡演出210场，新建乡村体育工程11个。加快党校新校区和老年大学建设。做好全国第一次可移动文物普查工作，扎实推进第二次全国地名普查。办好荆门国际马拉松赛、“光谷—农谷”自行车骑行赛、第七届市运会等大型赛事活动。

创新社会治理。加快法治城市创建，启动“七五”普法工作。健全预防和化解社会矛盾体系，完善律师参与信访矛盾化解工作机制。深化“三边警务”建设，严厉打击各类违法犯罪行为，打造“环荆护城河”。加强应急管理，提高防灾减灾救灾能力。全面落实安全生产责任和管理制度，坚决遏制各类重特大事故发生。加强食品药品安全监管，切实保障老百姓的食品安全、用药安全、生命安全。依法管理宗教事务，落实民族政策，推进援疆援恩工作。充分发挥工会、共青团、妇联、工商联、残联等群团组织在服务经济发展中的重要作用。进一步抓好保密、对台、档案、地方志、科普、统计、物价、外事侨务、地震、气象等各项工作。加强国防动员和后备力量建设，推进军民融合发展。

各位代表，在做好以上各项工作的同时，市政府将着力办好以下民生实事：

1.坚持整村推进，实现75个贫困村脱贫“销号”，1个贫困乡镇脱贫“摘帽”，6.03万名贫困人口脱贫。

2.全市城镇新增就业4.5万人，组织城乡劳动者就业培训1.2万人次。发放创业担保贷款5亿元，为4000名创业者提供融资服务。把2000名进城农民工和灵活就业人员纳入住房公积金保障体系。

3.完成42所义务教育学校标准化建设。新改建150个村卫生室，实现全市村卫生室“五化”达标率100%。

4.拉通农村“断头路”500公里，打通中心城区果园二路、果园四路、天鹅湖北路等“断头路”，畅通白龙山景区道路等微循环。新开通2条公交线路。

5.新建、改造1256个农村电网配电台区。解决10万农村居民饮水安全问题。

6.对3000名残疾人开展技术培训，为贫困残疾人配发辅助器具1500件。为困难群众办理法律援助案件1200件，其他援助事项12000件。

7.中心城区安装工地扬尘污染在线监控设施20套，推动10个规模较大的建筑工地安装喷洒降尘设施。

8.实施52个乡镇污水处理厂和24个村级污水处理设施建设工程。建设农村生活垃圾治理示范村300个，建成乡镇生活垃圾中转站20座、区域性生活垃圾处理厂3座。

9.新筹集公租房1497套、棚户区改造安置住房13461套。完成农村危房改造3000户。

10.对中心城区161个“三无”小区实施综合整治。对15家菜市场实施达标升级改造。

各位代表，确保“十三五”开好局、起好步，对政府自身建设提出了更新、更高的要求，我们将坚持以为民、务实、清廉和群众满意为核心，全面建设法治政府、服务型政府和廉洁政府。

坚持依法行政。推动全市各级政府及工作人员学法用法，启动政府规章制定工作，建立健全行政裁量权基准制度，强化重大执法决定法制审核，加强新型智库建设，严格遵循决策程序，做到行政行为实体合法、程序正当。主动接受市人大及其常委会的法律监督、工作监督和市政协的民主监督，认真办理人大代表议案、建议和政协提案，广泛听取各民主党派、工商联、无党派人士和各人民团体的意见。

坚持高效施政。坚决执行改进工作作风、密切联系群众的各项规定，巩固拓展“三严三实”专题教育成果。继续弘扬“抢、拼、严、实”的作风，积极主动作为，奋力争先进位。加大电子政务建设力度，推进信息共享和业务协同，提高政府运行效能和透明度。加强对政府系统工作落实情况的监督，扎实开展履职尽责明察暗访，治理为官不为，防止懒政怠政，惩处失职渎职。

坚持清廉从政。进一步落实政府党组党风廉政建设主体责任，扎实抓好政府系统廉政建设，着力形成不敢腐、不能腐、不想腐的长效机制。深入学习《中国共产党廉洁自律准则》和《中国共产党纪律处分条例》，强化公职人员廉洁从政意识。加强行政监察、审计和财政监督，深化政务公开，落实部门廉政建设监管责任。深入排查和防范重点领域、关键环节廉政风险，促进权力安全运行。加大惩贪治腐力度，营造风清气正氛围。

各位代表！回顾“十二五”，勤劳智慧的荆门人民创造了辉煌成就；展望“十三五”，跨越式发展的宏伟蓝图催人奋进。让我们紧密团结在以习近平同志为核心的党中央周围，在省委、省政府和市委的坚强领导下，高举旗帜，解放思想，开拓进取，真抓实干，为建成江汉平原中心城市、湖北新兴增长极、全国新型城镇化和城乡一体化示范区而努力奋斗！
